# Enhanced remineralization and acid resistance of white spot lesions by combined bioactive glass and resin infiltration: an in vitro study

**DOI:** 10.1186/s12903-026-08312-8

**Published:** 2026-04-10

**Authors:** Weibin Shen, Di Wu

**Affiliations:** 1 Key laboratory of Shaanxi Province for Craniofacial Precision Medicine Research, College of Stomatology, Xi’an Jiaotong University, Xi’an, Shaanxi China; 2 Clinical Research Center of Shaanxi Province for Dental and Maxillofacial Diseases, Xi’an Jiaotong University, Xi’an, Shaanxi China; 3 College of Stomatology, Xi’an Jiaotong University, Xi’an, Shaanxi China

**Keywords:** Bioactive glass, Resin infiltration, White spot lesions, Remineralization, Secondary demineralization, Surface microhardness, Surface roughness

## Abstract

**Objective:**

This study aimed to evaluate the combined and potentially synergistic effects of bioactive glass (BG) and resin infiltration (RI) on the remineralization and resistance to secondary demineralization of orthodontic white spot lesions (WSLs).

**Methods:**

One hundred twenty human premolars were randomly divided into four groups (*n* = 30 per group): RI group, BG group, combined RI + BG group, and untreated control group (NT). After creating artificial WSLs, specimens underwent a 4-week remineralization treatment followed by a 2-week secondary demineralization challenge. Surface roughness, microhardness, calcium ion release, and laser scanning confocal microscopy (LSCM) parameters were measured.

**Results:**

Post-remineralization, the RI + BG group showed the lowest surface roughness, highest microhardness, and most favorable LSCM parameters (TF, FA, AF), with significant differences compared to other groups (*p* < 0.001). Following secondary demineralization, the RI + BG group maintained the best performance, exhibiting the smallest changes in roughness and microhardness, and the least calcium ion release. The overall efficacy ranking was RI + BG > BG > RI > NT.

**Conclusion:**

The combination of bioactive glass and resin infiltration exhibited significantly superior remineralization efficacy and resistance to secondary demineralization compared to either treatment alone. This enhanced performance is supported by statistical evidence of synergistic interactions for key mechanical and chemical resistance outcomes, aligning with the observed superior overall effect.

## Introduction

White spot lesions (WSLs), characterized by subsurface enamel demineralization with a relatively intact surface layer, are a common complication during orthodontic treatment, affecting 46.7%–68.4% of patients within six months of fixed appliance placement [1]. While phosphoric acid etching prior to bracket bonding creates microporosities essential for resin-based adhesive retention [2], this iatrogenic surface roughness promotes cariogenic biofilm accumulation, leading to pathological demineralization [3]. Conventional preventive measures, such as fluoride varnish and casein phosphopeptide-amorphous calcium phosphate (CPP-ACP), achieve only limited WSL regression due to insufficient lesion penetration [4], highlighting the need for more effective remineralization strategies.

Resin infiltration (RI) has emerged as a promising microinvasive treatment. It utilizes a low-viscosity infiltrant to occlude enamel pores via capillary action after mild acid etching, thereby stabilizing the lesion and reducing further demineralization [5]. Clinical studies report that RI can reduce lesion progression by 37.2% at 18 months [6]. However, a significant proportion of RI-treated lesions (22.7%) may experience secondary demineralization at the resin-enamel interface due to incomplete pore sealing and biofilm adhesion [5].

Bioactive glass (BAG), particularly 45S5 composition, offers an alternative approach through distinct remineralization kinetics. Upon contact with oral fluids, BAG releases calcium and phosphate ions, forming hydroxycarbonate apatite that integrates with enamel crystallites, thereby increasing microhardness [7]. Additionally, BAG’s alkaline pH elevation upon dissolution exhibits antimicrobial effects against cariogenic bacteria like *Streptococcus mutans* [8].

However, both RI and BAG exhibit inherent limitations when used alone. RI primarily provides a superficial seal but may not fully remineralize the lesion body and is susceptible to secondary demineralization at the interface [5]. Conversely, BAG promotes profound remineralization but lacks the immediate ability to create a robust physical barrier against acid attack. Given the complementary mechanisms of RI (physical pore sealing) and BAG (chemical remineralization and antimicrobial action), their combined application may yield synergistic benefits. However, the existing literature lacks conclusive evidence on whether the sequential application of BAG and RI truly produces a synergistic effect that surpasses the efficacy of either treatment alone. Furthermore, it remains unclear whether such a combined strategy can confer enhanced long-term stability and resistance against secondary acid challenges, which is a critical clinical consideration for managing WSLs. To address this knowledge gap, this study specifically investigates the efficacy of combined bioactive glass and resin infiltration treatment on the remineralization kinetics and resistance to secondary demineralization of orthodontic white spot lesions, and evaluates potential synergistic interactions through statistical modeling. The null hypothesis is that no significant differences exist in remineralization efficacy or acid resistance between BAG monotherapy, RI monotherapy, and combined BAG-RI treatment under pH-cycling conditions.

## Materials and methods

### Ethical approval

This in vitro study utilized extracted human teeth. The study protocol was approved by the Ethics Committee of the College of Stomatology, Xi’an Jiaotong University (Approval No. 2024-0028). The requirement for informed consent was waived as the teeth were anonymized specimens discarded as medical waste. All procedures adhered to the principles of the World Medical Association’s Declaration of Helsinki.

### Sample size determination

A priori power analysis was conducted using G*Power 3.1 software (Heinrich-Heine-Universität Düsseldorf, Germany) [9]. Based on a pilot study of enamel microhardness recovery (ΔSMH) comparing bioactive glass (BAG) vs. control groups (*n* = 10 per group), an effect size (*d*) of 0.52 was calculated using Cohen’s *d* formula [10]:

1 $$\:d=\frac{{\overline{x}}_{1}-{\overline{x}}_{2}}{{s}_{pooled}}$$

2 $$\:{s}_{pooled}=\sqrt{\frac{\left({n}_{1}-1\right){s}_{1}^{2}+\left({n}_{2}-1\right){s}_{2}^{2}}{{n}_{1}+{n}_{2}-2}}$$

With *α* = 0.05 (two-tailed), power (1-*β*) = 0.80, and allocation ratio 1:1 for four experimental groups, the analysis indicated a minimum requirement of 23 specimens per group for one-way ANOVA [11]. To account for potential attrition during pH-cycling (estimated 20% loss from enamel fracture) [12] and enhance statistical robustness, the final sample size was increased to 30 per group (total *N* = 120).

### Sample preparation

A total of 120 recently extracted human premolars were selected. The selection criteria were [13]: normal tooth development, healthy, without caries, cracks, and pigments. The teeth were cleaned and stored in 0.9% sodium chloride solution. The roots were sectioned, and the buccal surfaces were polished sequentially with silicon carbide paper (800, 1000, 1200, 2400 grit). A 4 mm × 4 mm window was created on the buccal surface of each tooth, and the surrounding areas were coated with acid-resistant nail polish [14].

### Establishment of demineralization model and experimental grouping

The demineralization protocol, including the solution composition, was established based on a classic pH-cycling model [15] with modifications. The solution had the following composition: 2.2 mmol/L CaCl₂·2 H₂O, 2.2 mmol/L NaH₂PO₄, 50 mmol/L acetic acid, pH 5. Fresh solution was prepared daily and verified by pH meter (Mettler Toledo FE28). The specimens were placed in an artificial demineralization solution (pH 5.0) in a 37 °C electric constant temperature water bath for demineralization, and the solution was shaken at a fixed mode of 100r/min. The artificial demineralization solution was replaced regularly every day to maintain the same pH value. After 2 weeks, demineralization was completed, and the samples were rinsed with a large amount of deionized water and air-dried at room temperature (25 °C) for 24 h in a dust-free environment. To minimize operator bias during subsequent measurements, the investigator performing the profilometry and microhardness tests was blinded to group allocation. The window area showed an obvious chalky color change, indicating completion of sample preparation. The depth of the artificial white spot lesions was verified in a pilot subset of samples (*n* = 5) by cross-sectional LSCM, confirming a consistent lesion depth within the range of 80–120 μm.

Enamel specimens (*N* = 120) were assigned unique identification codes (S001-S120) and stratified by pre-demineralization microhardness values (320–350 Vickers Hardness Number, VHN), then randomly allocated into four equal groups (*n* = 30 per group) using a computer-generated sequence produced by Random Allocation Software 2.0 with a fixed seed number (20230508) and block size of 4, where allocation concealment was maintained through sequentially numbered opaque sealed envelopes. The four experimental groups consisted of: the BG group treated with 45S5 bioactive glass slurry (10% w/v, 5 min application) [16]; the RI group receiving resin infiltrant (Icon^®^, DMG) with 15% hydrochloric acid etching; the BG + RI group subjected to the following sequential treatment: First, the bioactive glass slurry (10% w/v) was applied for 5 min, then gently rinsed off with deionized water and air-dried, Subsequently, the specimen was etched with 15% hydrochloric acid gel (Icon Etch, DMG) for 120 s as per manufacturer’s instructions, thoroughly rinsed with deionized water for 30 s, and dried, Finally, the resin infiltrant (Icon^®^, DMG) was applied and light-cured; and the NT group serving as an untreated control immersed in artificial saliva. All groups underwent identical remineralization conditions. Artificial saliva was replaced every 48 h for all specimens to ensure consistent ion availability throughout the 4-week remineralization period. The artificial saliva [15] contained 0.70 g/L NaCl, 1.20 g/L KCl, 0.294 g/L CaCl₂·2 H₂O (providing 2.0 mmol/L Ca²⁺), 0.136 g/L KH₂PO₄ (providing 1.0 mmol/L PO₄³⁻), and 6.06 g/L Tris (50 mmol/L buffer), with the pH adjusted to 7.20 ± 0.05 using 1 mol/L HCl at 37 °C.

### Secondary demineralization treatment

Following the 4-week remineralization period, all sample groups underwent a 2-week secondary demineralization treatment to assess acid resistance of remineralized lesions [17]. Samples were immersed in demineralizing solution (pH 5.0) maintained at 37 °C in a thermostatic water bath, with daily solution replacement to ensure consistent demineralization kinetics. Post-treatment, specimens were rinsed with deionized water and stored in artificial saliva. Demineralization solutions from Day 1, 7, and 14 of immersion were collected in sterile sealed tubes for analysis.

### Measurement surface roughness

Surface roughness measurements of dental enamel specimens were performed using a TR220 profilometer (Time Group Inc., China) equipped with a custom ultra-fine stylus (tip radius ≤ 2 μm) and force-calibrated module (vertical force ≤ 4 mN) to prevent surface damage. Measurements were performed with the stylus traversing perpendicular to the enamel prism direction to ensure consistency. All assessments were conducted in a controlled environment (temperature: 25 ± 1 °C, relative humidity: 50 ± 5%). Measurements were conducted at four stages: pre-demineralization, post-demineralization, post-remineralization treatment, and post-secondary demineralization treatment. Measurement parameters were set as follows: scanning speed 0.2 mm/s, Gaussian filter with cut-off wavelength *λc* = 0.25 mm (ISO 4287), and evaluation length 1.25 mm (5 × sampling length *Lc* = 0.25 mm). At each stage, five randomly selected locations per sample were measured avoiding cracks or pores, with mean Ra (arithmetic average roughness) values reported for comparative analysis [17].

### Measurement of surface microhardness (SMH)

Vickers microhardness testing was performed using a digital microhardness tester (Wilson Tukon 1102, Buehler Ltd., USA) with a diamond pyramid indenter (136° apex angle per ISO 6507-1) under a 150 gf load for a 15 s dwell time, Indentations were systematically positioned in a standardized cruciform pattern centered on the lesion zone, comprising a primary indentation at the specimen’s geometric center (4 × 4 mm² surface) and four secondary indentations at 200 μm intervals along orthogonal axes (± 100 μm from center). This ensured spatial consistency across all measurement phases (pre-demineralization, post-demineralization, post-remineralization, post-secondary demineralization) while maintaining ≥ 3 × indentation diagonal spacing to prevent plastic deformation interactions. Diagonal lengths (*d₁*,* d₂*) were measured at 400 × magnification (Olympus BX51M), with Vickers hardness numbers (VHN) calculated as *VHN* = 1.8544 × *P* / *d²* (*P* = load in kgf, *d* = mean diagonal length in mm). Five valid measurements per specimen were averaged after excluding indentations within 50 μm of surface defects as pre-screened by confocal laser scanning. Intra-group consistency was confirmed by a mean coefficient of variation (CV) of < 5% across replicate indentations. Indentations with blurred edges (occasionally observed in severe WSLs) were excluded, and a replacement measurement was taken at an adjacent standardized location.

### Sequential measurement protocol and spatial considerations

Both surface roughness (Ra) and surface microhardness (SMH) measurements were performed on the same set of specimens (*N* = 120) in a sequential, non-destructive manner. To prevent interference between the two measurement techniques, a strict spatial separation protocol was followed. Profilometry was always performed first using an ultra-fine stylus (tip radius ≤ 2 μm, force ≤ 4 mN) to ensure non-destructive scanning. Subsequently, Vickers indentations were placed at least 500 μm away from any profilometry scan path and from adjacent indentations, ensuring that plastic deformation zones did not affect surface topography. This distance was more than five times the indentation diagonal length (typically 50–100 μm), guaranteeing the independence and integrity of both datasets. This protocol ensured that the plastic deformation zone of the hardness indentations did not affect the surface topography measured by the profilometer, and vice versa, thereby maintaining the integrity and independence of both datasets.

### Determination of calcium ion deposition

During secondary demineralization (Days 1, 7, and 14), 1 ml of demineralized solution was collected from each sample. Samples were protected from light and stored at 4 °C, with calcium ion concentration measured within 24 h using a colorimetric method. The assay was performed with o-cresolphthalein complexone (OCPC) chromogenic reagent (Cat. No. C7564, Sigma-Aldrich) on a Beckman AU5800 fully automated biochemical analyzer. Samples were analyzed undiluted. Internal quality control was performed using low (0.8 mmol/L), medium (2.0 mmol/L), and high (4.0 mmol/L) calcium standards (Randox Laboratories) in each run, with all results falling within ± 5% of the target values. Calibration curves were established using calcium standard solutions (0.5-5.0 mmol/L, Randox Laboratories) at 570 nm wavelength, with a sensitivity of 0.05 mmol/L. Triplicate measurements were conducted per sample group, and mean values were reported.

### Laser scanning confocal microscope (LSCM) imaging scanning

In a light-proof environment, a Rhodamine B fluorescent dye solution (0.1 mmol/L) was prepared. Specimens from each group—pre-demineralization, post-demineralization, and post-remineralization—were immersed in the dye solution and incubated at 37 °C in a constant-temperature water bath for 2 h. Following incubation, the specimens were thoroughly rinsed with copious amounts of deionized water to remove excess, non-specifically adsorbed dye from the surface, thereby minimizing background fluorescence and preventing obscuration of the true lesion signal during LSCM observation. Finally, the samples were gently air-dried prior to imaging to obtain a stable and clear observation surface. An OLYMPUS FLUOVIEW FV1000 laser scanning confocal microscope was used to scan the samples layer by layer at a depth of 20 to 50 μm to observe the fluorescence penetration (the red fluorescent area indicates the demineralized area). Images were collected on a computer, and the fluorescent area (FA), average fluorescence (AF), and total fluorescence (TF) were measured [18, 19]. Images were analyzed using FV10-ASW 4.2 software (Olympus). The demineralized area was segmented by applying a threshold based on fluorescence intensity (> 50% of the maximum). Fluorescent Area (FA) was calculated as the total pixel count of the thresholded region multiplied by the pixel area (µm²). Average Fluorescence (AF) was computed as the mean intensity per pixel within the thresholded region. Total Fluorescence (TF), representing the integrated optical density of the lesion, was calculated by the software as the sum of the intensity values of all pixels within the thresholded region.

### Statistical analysis

All quantitative data were expressed as mean ± standard deviation (SD) with 95% confidence intervals (CI). Normality was assessed separately for each variable at each time point (pre-demineralization, post-demineralization, post-remineralization, post-secondary demineralization) using the Shapiro-Wilk test (α = 0.05), and homogeneity of variances was verified via Levene’s test.

For normally distributed data with homogeneous variances (surface roughness, microhardness, LSCM parameters), both one-way ANOVA (for four-group comparisons) and 2 × 2 factorial ANOVA were performed. The factorial ANOVA included ‘Bioactive Glass’ (absent/present) and ‘Resin Infiltration’ (absent/present) as independent factors, with their interaction term (BG × RI) tested to assess synergy. Post-hoc pairwise comparisons used the Games-Howell test.

For calcium ion concentration across three time points (Days 1, 7, 14), a Two-Way Mixed ANOVA (Group × Time) was employed to evaluate interaction and main effects. Due to violations of normality at some time points, non-parametric Kruskal-Wallis H tests with Cliff’s Delta effect size (95% CI via bootstrap) were also conducted at each time point to ensure robust inter-group comparisons.

For all other data violating parametric assumptions, non-parametric tests were used: Kruskal-Wallis H test for inter-group comparisons, Friedman test for intra-group longitudinal changes, with respective post-hoc procedures (Dunn’s test, Wilcoxon signed-rank test with Bonferroni correction). The significance threshold was set at *p* < 0.05. All analyses were performed using IBM SPSS Statistics (version 28.0).

## Results

### Enamel surface roughness

Two-way ANOVA revealed a significant interaction between BG and RI treatments on post-remineralization surface roughness (*F* (1, 116) = 558.87, *p* < 0.001, *η²* = 0.83), indicating a synergistic interaction. Statistical analysis revealed significant differences inter- and intra-group comparisons. In inter-group comparisons, group RI + BG demonstrated superior performance in both post-remineralization and post-secondary demineralization. Post-remineralization, the surface roughness value of group RI + BG was significantly lower than that of group RI (mean difference Δ = 2.229, 95% CI [2.217–2.242]; *p* < 0.001), group BG (Δ = 1.031, 95% CI [1.015–1.047]; *p* < 0.001), and untreated control group NT (Δ = -4.049, 95% CI [– 4.190–3.909]; *p* < 0.001). This advantage persisted post-secondary demineralization, with mean differences of 2.630 (vs. RI), 1.310 (vs. BG), and – 4.049 (vs. NT) (*p* < 0.001 for all). Intra-group comparisons highlighted distinct mineralization dynamics. While all groups exhibited significant changes between post-remineralization and post-secondary demineralization (*p* < 0.001), group RI + BG displayed the smallest variation (Δ = 0.0797, *t* = 14.0689), contrasting sharply with the largest instability observed in the control group NT (Δ = 1.2500, *t* = 11.2464). Notably, group RI, BG, and RI + BG showed progressive reductions in mineral loss post-secondary demineralization (mean differences: – 0.2887, – 1.6297, – 2.9490; *p* < 0.0024), whereas group NT exhibited paradoxical mineral depletion (Δ = 1.1200, *p* < 0.001) (see Table 1).

**Table 1:** Surface roughness analysis of each group before and after enamel treatment. $\:\left(\stackrel{-}{x}\pm\:s,\:\:n=30\right)$

Group	pre-demineralization	post-demineralization	post-remineralization	post-secondary demineralization
RI	2.88 ± 0.53	3.27 ± 0.93	2.50 ± 0.05^bd^​	2.98 ± 0.16^bd*^
BG	2.79 ± 0.85	3.29 ± 0.85	1.30 ± 0.0 6^ad^	1.66 ± 0.09^ad*^
RI + BG	2.83 ± 0.81	3.30 ± 0.80	0.27 ± 0.04^abd^	0.35 ± 0.05^abd*^
NT	2.83 ± 0.59	3.28 ± 0.89​	3.15 ± 0.75	4.40 ± 0.70^*^​

Note: ① Lowercase letters represent pairwise comparisons between groups, and groups with different letters have *p* < 0.05 ; ② * indicates *p* < 0.05 compared with the post-demineralization. Group RI: ICON infiltration resin group; Group BG: bioactive glass group; Group RI + BG: ICON infiltration resin + bioactive glass group; Group NT: blank control group

### Enamel surface microhardness value (SMH)

A significant interaction between BG and RI was observed for post-remineralization SMH (*F* (1, 116) = 572.81, *p* < 0.001, *η²* = 0.83), confirming a synergistic effect on enamel mechanical recovery. Statistical analysis demonstrated significant inter- and intra-group differences. In inter-group comparisons, group RI + BG exhibited superior performance in both post-remineralization and post-secondary demineralization. Post-remineralization, the SMH value of group RI + BG was significantly higher than that of group RI (Δ = – 43.14, 95% CI [– 44.72, – 41.56]; *p* < 0.001), group BG (Δ = – 20.10, 95% CI [– 21.65, – 18.55]; *p* < 0.001), and group NT (Δ = 79.10, 95% CI [77.63, 80.57]; *p* < 0.001). This advantage persisted post-secondary demineralization, with larger differences versus group RI (Δ= – 94.10), group BG (Δ = – 42.10), and group NT (Δ = 154.60; all *p* < 0.001). Intra-group comparisons revealed distinct mineralization dynamics: all groups experienced significant changes between remineralization and secondary demineralization(*p* < 0.001), but group RI + BG showed the smallest decline (Δ = – 28.30, *t* = – 32.27), contrasting sharply with group NT’s severe depletion (Δ = – 103.80, *t* = – 78.00). Comparisons with pre-demineralization values further highlighted group RI + BG’s residual protection (post-demineralization Δ = 67.80 vs. pre-demineralization), whereas groups RI and NT exhibited net mineral loss (Δ = – 24.80/– 81.66) (see Table 2).

**Table 2:** SMH before and after enamel treatment in each group $\:\left(\stackrel{-}{x}\pm\:s,\:\:n=30\right)$

Group	pre-demineralization	post-demineralization	post-remineralization	post-secondary demineralization
RI	318.20 ± 4.61	205.20 ± 6.85	260.20 ± 4.43 ^bd^	180.90 ± 5.55^bd*^
BG	318.10 ± 5.40	201.00 ± 6.92	283.20 ± 3.88 ^ad^	232.90 ± 7.55^ad*^
RI + BG	321.50 ± 3.34	207.20 ± 5.15	303.30 ± 6.33^abd^	275.00 ± 5.99^abd*^
NT	318.10 ± 4.10	202.00 ± 8.34	224.20 ± 3.26	120.40 ± 5.47^*^

Note:① Lowercase letters represent pairwise comparisons between groups, and groups with different letters have *p* < 0.05; ② * indicates *p* < 0.05 compared with the post-demineralization. Group RI: ICON infiltration resin group; Group BG: bioactive glass group; Group RI + BG: ICON infiltration resin + bioactive glass group; Group NT: blank control group

### Measurement of calcium ion precipitation

The two-way mixed ANOVA revealed a significant Group × Time interaction for calcium ion concentration (*F* (6, 232) = 212.33, *p* < 0.001, *η²* = 0.846). Greenhouse-Geisser correction was applied due to violation of sphericity (Mauchly’s *W* = 0.50, *p* < 0.001). The large effect size indicates that the combined treatment (RI + BG) conferred a synergistically enhanced resistance to acid challenge over time. The intergroup analysis revealed non-normal distribution (Shapiro-Wilk test, *α* = 0.05) and heteroscedasticity (Levene’s test, *p* < 0.001), necessitating the Kruskal-Wallis non-parametric test. Significant differences were observed across all timepoints (*p* < 0.001). Dunn’s post hoc test with Bonferroni correction demonstrated that group RI + BG exhibited extreme significance compared to groups RI and NT at 1d, 7d, and 14d (1d vs. RI: *p* = $\:1.57\times\:{10}^{-9}$, vs. NT: *p* = $\:2.63\times\:{10}^{-21}$; 14d vs. NT: *p* = $\:5.60\times\:{10}^{-23}$), with the most pronounced disparity against group NT. Bootstrap-derived 95% confidence intervals for median differences confirmed progressively increasing effect sizes between group RI + BG and others (1d: [–0.325, – 0.27], Cliff’s Delta > 0.7; 14d: [– 0.910, – 0.875], |Δ| = 1), validating clinically meaningful distinctions.

Intragroup temporal analysis using the Friedman test (*p* < 0.05) and Wilcoxon signed-rank tests identified significant sequential changes in groups RI and RI + BG (7d-1d: *p* = $\:1.70\times\:{10}^{-6}$; 14d-7d: *p* = $\:1.71\times\:{10}^{-6}$). Notably, group RI + BG showed no significant variation between 7d and 14d (*p* = 0.146), indicating stable anti-demineralization efficacy. In contrast, paired t-tests revealed divergent patterns: group BG exhibited no 14d-7d difference (mean Δ = -0.006, *p* = 0.940, 95% CI [– 0.019, 0.017]), while group NT demonstrated sustained escalation, with a 14d-1d mean increase of 0.611 (95% CI [0.587, 0.634], *p* = $\:1.67\times\:{10}^{-30}$). These results underscore fundamentally distinct regulatory mechanisms of calcium ion dynamics across intervention protocols. As summarized in Table 3.

**Table 3:** Comparison of calcium ion precipitation results in each group $\:\left(\stackrel{-}{x}\pm\:s,\:\:n=30\right)$

Group	post-secondary demineralization 1d	post-secondary demineralization 7d	post-secondary demineralization 14d
RI	1.35 ± 0.06 ^bd^	1.50 ± 0.06 ^bd *^	1.61 ± 0.05^bd*#^
BG	1.13 ± 0.09 ^ad^	1.38 ± 0.09 ^ad *^	1.38 ± 0.07^ad *^
RI + BG	1.05 ± 0.07^abd^	1.25 ± 0.07^abd *^	1.24 ± 0.01^abd *^
NT	1.52 ± 0.05	1.98 ± 0.11 ^*^	2.13 ± 0.10^*#^

Note: * indicates *p* < 0.05 compared with the 1st day ; ② # indicates *p* < 0.05 compared with the 7th day ; ③ Lowercase letters indicate pairwise comparisons between groups, and *p* < 0.05 between groups with different letters. Group RI: ICON infiltration resin group; Group BG: bioactive glass group; Group RI + BG: ICON infiltration resin + bioactive glass group; Group NT: blank control group

### LSCM images

**Fig. 1:**
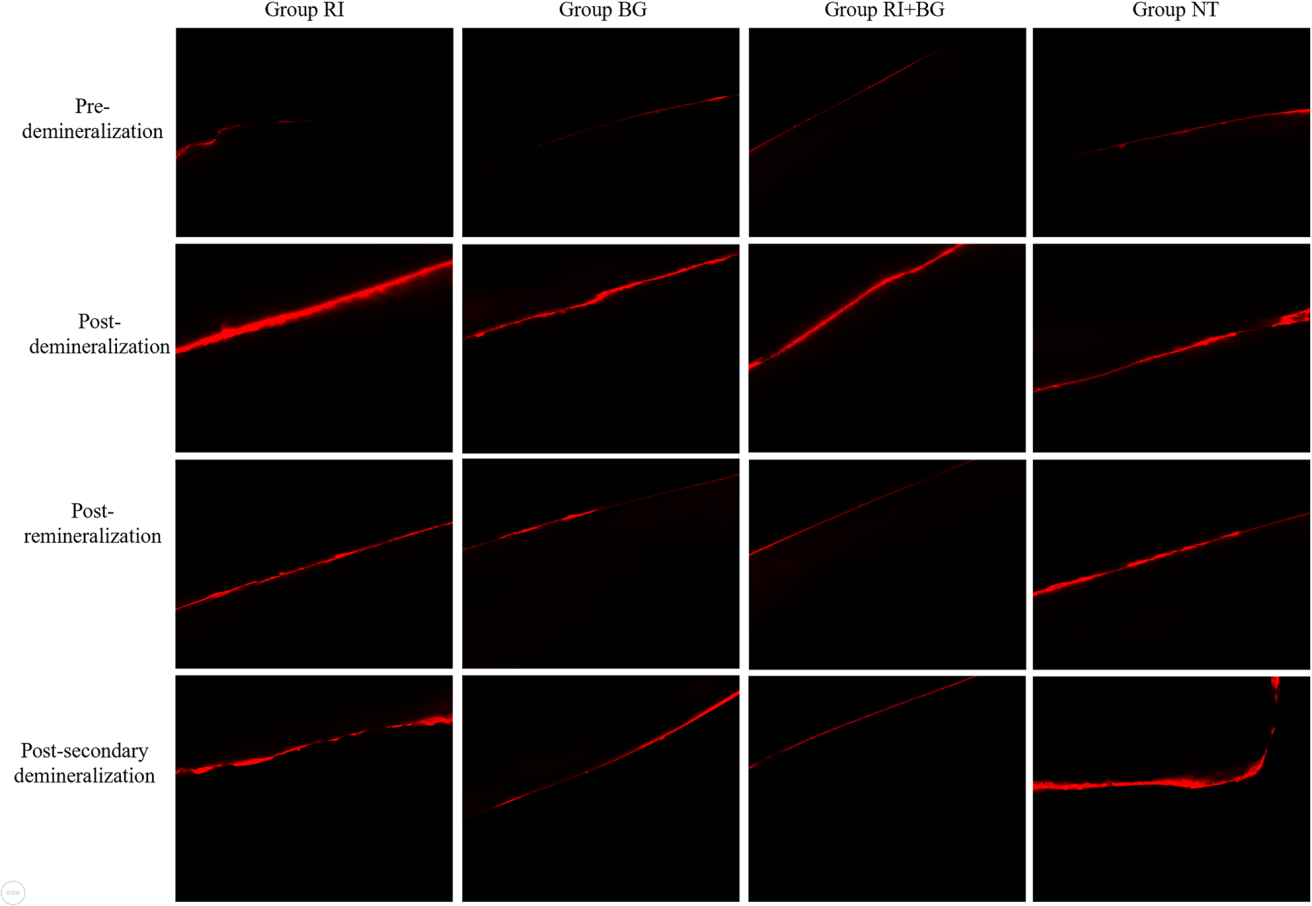
LSCM image. Note: Representative laser scanning confocal microscopy (LSCM) images of enamel surfaces in each group at four experimental stages: pre-demineralization, post-demineralization, post-remineralization, and post-secondary demineralization. Red fluorescence indicates demineralized areas. After remineralization, the RI + BG group exhibited the narrowest and darkest fluorescence bands, indicating the highest degree of enamel structure restoration. Following the secondary demineralization challenge, the RI + BG group maintained the most favorable appearance, with fluorescence bands remaining narrow and faint, demonstrating superior long-term acid resistance. The BG group showed moderate widening of fluorescence bands, while the RI group displayed more pronounced fluorescence, suggesting marginal leakage at the resin-enamel interface. The untreated control (NT) group exhibited the broadest and brightest fluorescence, reflecting progressive demineralization. All images were acquired under identical magnification and scanning parameters. Scale bar = 100 μm applies to all panels. Group RI: ICON infiltration resin group; Group BG: bioactive glass group; Group RI + BG: ICON infiltration resin + bioactive glass group; Group NT: blank control group

Laser scanning confocal microscopy was used to detect changes in fluorescence bands on the same observation surface across four experimental stages. Pre-demineralization, the enamel surfaces of all four groups were smooth and flat, with similar fluorescence band brightness and width, and no notable differences observed between groups. Post-demineralization, the enamel surfaces of all groups appeared chalky and rough, and the fluorescence bands became brighter and wider, with no distinction between groups, confirming successful lesion creation. Post-remineralization, the width and brightness of fluorescence bands in all treatment groups decreased compared to post-demineralization, indicating varying degrees of enamel repair. The RI and NT groups exhibited slightly rough surfaces with relatively higher fluorescence band width and brightness. The BG and RI + BG groups displayed flatter enamel surfaces with narrower and darker fluorescence bands. Among them, the RI + BG group showed the narrowest and darkest fluorescence bands, closest to the pre-demineralization state, indicating the highest degree of remineralization and near-normal enamel structure restoration. Post-secondary demineralization, the RI + BG group maintained the most favorable appearance, with fluorescence bands remaining narrow and faint, demonstrating superior long-term acid resistance. The BG group showed moderate widening of fluorescence bands, while the RI group displayed more pronounced fluorescence, suggesting marginal leakage at the resin-enamel interface. The untreated control (NT) group exhibited the broadest and brightest fluorescence, reflecting progressive demineralization (Fig. 1).

### Fluorescence results of each group after remineralization

Post-demineralization, the mineral content in the enamel decreased, enamel microporosity increased, and the red fluorescent dye filled the pores. The total fluorescence (i.e., total fluorescence), fluorescence depth (i.e., average fluorescence) and demineralized area (i.e., fluorescence area) of the demineralized area (red area) were measured using laser confocal scanning microscopy to evaluate demineralization and remineralization of the enamel surface.

For the LSCM parameters (TF, FA, AF), the combined RI + BG treatment yielded the most favorable results. However, the interaction term in the two-way ANOVA was not statistically significant (*p* > 0.05). Statistical results demonstrated significant between-group differences in TF (*F* (3, 56.36) = 238.92, *p* < 0.001, *η²* = 0.842), FA (*F* (3, 62.77) = 253.11, *p* < 0.001, *η²* = 0.869), and AF (*F* (3, 63.92) = 144.24, *p* < 0.001, *η²* = 0.793), with effect sizes indicating that experimental interventions accounted for 84.2%, 86.9%, and 79.3% of total variance, respectively. Games-Howell post-hoc tests revealed statistically significant differences (*p* < 0.001) between group RI + BG and control groups. For TF, the mean differences compared to groups RI, BG, and NT were 2.410 (95% CI: 2.168 to 2.652), 1.190 (95% CI: 0.920 to 1.460), and 4.859 (95% CI: 4.369 to 5.350), respectively. For FA, the corresponding differences were 2.610 (95% CI: 2.374 to 2.855), 1.130 (95% CI: 0.842 to 1.419), and 4.670 (95% CI: 4.268 to 5.073). For AF, the differences were 30.722 (95% CI: 25.334 to 36.111), 19.709 (95% CI: 14.503 to 24.915), and 49.438 (95% CI: 44.376 to 54.499). All confidence intervals excluded zero with *p*-values < 0.001, confirming group RI + BG’s robust intervention effects across all three metrics and the statistical reliability of between-group differences. As summarized in Table 4.

**Table 4:** TF (×10^4^), FA (×10^2^ µm^2^), AF of each group $\:\left(\stackrel{-}{x}\pm\:s,\:\:n=30\right)$

Group	TF	FA	AF
RI	5.54 ± 0.91^bd^	6.98 ± 1.04^bd^	161.13 ± 16.47^bd^
BG	4.32 ± 1.13^ad^	5.51 ± 1.21^ad^	150.05 ± 15.00^ad^
RI + BG	3.13 ± 0.57^abd^	4.38 ± 0.95^abd^	130.41 ± 19.52^abd^
NT	7.99 ± 2.32	9.05 ± 1.81	179.85 ± 13.76

Note: Fluorescence parameters of each group after remineralization. Lowercase letters represent pairwise comparisons between groups, and groups with different letters have *p* < 0.05. Group RI: ICON infiltration resin group; Group BG: bioactive glass group; Group RI + BG: ICON infiltration resin + bioactive glass group; Group NT: blank control group

*TF* total measurement fluorescent, *FA* fluorescent area, *AF* average fluorescent

## Discussion

During or after orthodontic treatment, the tooth surface generally changes to a chalky white color, which is called white spot lesions (WSLs) [20]. When demineralization develops to the late stage, the demineralization rate is much faster than the remineralization rate, and the remineralization effect is minimal. The defect can only be treated by tooth restoration. Therefore, the treatment of early enamel demineralization is necessary to stop the progression of caries, preserve as much remaining tooth structure as possible, and restore the aesthetic appearance of the tooth.

Due to its high fluidity and good penetration, infiltration resin can penetrate into the micropores of early caries through a capillary action mechanism, build a protective layer, block the entry of acidic substances, fill the damaged enamel, prevent the internal structure from collapsing, and stabilize its structure [21]. Some studies have shown that surface infiltration resin can repair demineralized areas, restore tooth color, and reverse the damage caused by demineralization over a brief duration. Its aesthetic effect is superior to that of traditional remineralization agents such as fluoride varnish [22, 23]. However, the penetration depth of infiltration resin only accounts for 75%-80% of enamel demineralization depth, and it cannot completely fill the surface layer of demineralized enamel defects [24, 25]. The findings of this research indicated that after treatment of demineralized enamel with infiltration resin alone, its surface microhardness increased and the roughness value decreased significantly; however, after secondary demineralization challenge, the surface microhardness value decreased significantly and the roughness value increased significantly, with the fluctuation range being second only to the control group. This indicates that using infiltration resin alone has certain limitations in achieving long-term stability and preventing re-demineralization of enamel, which aligns with the findings of Mandava et al. [26].

Bioactive glass (BAG) is an active material whose main components are SiO2, Na_2_O, CaO, P_2_O_5_, etc. Its solution has strong permeability and can penetrate deep into the demineralized enamel to form a hydroxyapatite-like structure, promote bone regeneration, improve acid resistance, and has good biocompatibility. In the last couple of years, it has been extensively applied in dentistry [27–29]. Its mechanism of action is as follows: after contact with saliva, the sodium ions in the BAG particles react biochemically with the hydrogen ions present in the saliva, leading to an increase in pH value, accelerating the precipitation of Ca and P ions, and depositing on the tooth surface together with the free calcium and phosphorus in the saliva to form basic calcium phosphate, thereby inducing the remineralization of demineralized enamel [30]. In this experiment, the combination of infiltration resin and BAG was used to treat the demineralization model. The RI + BG group demonstrated significantly higher SMH than both monotherapy groups post-remineralization (Δ = 20.10 VHN vs. BG, Δ = 43.14 VHN vs. RI; *p* < 0.001 for both). This advantage was further amplified following secondary acid challenge (Δ = 42.10 VHN vs. BG, Δ = 94.10 VHN vs. RI; *p* < 0.001). It was found that the roughness value of group RI + BG was the smallest, indicating that infiltration resin combined with bioactive glass can better restore the surface smoothness of demineralized enamel, and its remineralization effect is superior to that of either the infiltration resin group or bioactive glass group alone. This may be because the bioactive glass fills the pores missed by the infiltration resin. Critically, the observed superior performance suggests an interaction that exceeds a simple additive or pore-filling effect.

Although the NT group received no active treatment, a slight improvement in surface microhardness and roughness was observed after the 4-week remineralization period. This is likely attributable to the presence of calcium and phosphate ions in the artificial saliva, which may have promoted limited spontaneous remineralization, as previously reported in pH-cycling models [15]. However, the degree of improvement in the NT group was significantly lower than that in all treatment groups (*p* < 0.001), confirming that the observed effects in the RI, BG, and RI + BG groups were primarily due to the active interventions rather than the storage medium alone.

In this experiment, the amount of calcium ion precipitation in the demineralization solution was measured at 1, 7, and 14 days. It was found that the amount of calcium ion dissolution in groups RI and NT increased with the extension of demineralization time, while there was no substantial alteration in the amount of calcium ion dissolution in groups BG and RI + BG at 7 and 14 days, suggesting that the efficacy of these two groups after remineralization was stable. The amount of calcium ion precipitation in the demineralization solution of group RI + BG was the least, and there was a significant decreasing trend, indicating that the remineralization effect of infiltration resin combined with bioactive glass was better than that of single use of bioactive glass. The amount of calcium ion precipitation in each group was NT > RI > BG > RI + BG. The above analysis shows that the stability after treatment with infiltration resin combined with bioactive glass is better, and the effect of inhibiting re-demineralization is the best.

LSCM images show a red fluorescent band, indicating the enamel demineralization area. Post-remineralization, the fluorescence intensity in each group ranked as RI + BG < BG < RI < NT, indicating that the combined use of ICON infiltration resin and BAG achieved the best remineralization effect and color restoration. Quantitative analysis of LSCM parameters (Table 4) confirmed this observation, with the RI + BG group exhibiting the most pronounced reduction in FA, AF, and TF compared to the other groups (*p* < 0.001). This suggests that the combined treatment achieved the most complete restoration of enamel structure. The moderate improvement in the RI group can be attributed to surface pore occlusion by the infiltrant, while the BG group demonstrated deeper remineralization, consistent with its ion-releasing mechanism. The superior performance of the RI + BG group reflects a complementary effect: BG-mediated intralesional mineral deposition followed by resin sealing, which together restored enamel integrity more effectively than either treatment alone. These findings align with the surface microhardness and roughness data, underscoring the complementary roles of chemical and physical remineralization strategies. Furthermore, the enamel surface of the RI + BG group exhibited more uniform mineral deposition and greater smoothness than the other groups [31]. In addition to the influence of calcium and phosphorus ions, bioactive glass also relies on the silicon ions it contains when promoting enamel remineralization. Studies [32, 33] have shown that silicon has a strong effect on promoting mineralization. The original crystal nucleus forms a solid crystal core containing calcium and phosphorus components with the participation of Si, further absorbing the surrounding equilibrium ions, so that the demineralized structure in the enamel is reorganized and reconstructed, accelerating remineralization. As a new type of remineralization material, its efficacy is better than that of traditional remineralization agents [34]. Some studies have shown that the remineralization degree of bioactive glass at a concentration of 60 g/L is higher than that at other concentrations [8]. Therefore, this experiment selected 60 g/L bioactive glass combined with resin to treat teeth with early enamel demineralization, and explored the efficacy of the combined treatment of early enamel demineralization.

Statistical analysis confirmed significant synergistic interactions (*p* < 0.001) for surface roughness, microhardness, and calcium ion resistance—key indicators of mechanical integrity and long-term chemical stability. This synergy likely stems from a sequential mechanism: BG-induced intralesional mineral precipitation is subsequently protected and stabilized by the RI sealant, creating a composite barrier with resistance to acid penetration that exceeds the sum of its parts.

Furthermore, a pertinent consideration in our sequential protocol is the potential chemical interaction between the applied BAG and the subsequent HCl etchant. BAG elevates pH via ion exchange, while HCl is a strong acid. It is plausible that residual BAG particles partially dissolved upon contact with the acid, exerting a local buffering effect and modulating the actual etching intensity at the enamel interface. This interaction may not diminish the etching efficacy but rather could promote a more uniform substrate by preventing over-etching, potentially enhancing the subsequent wetting and penetration of the resin infiltrant. Although not directly quantified in this study, such an interfacial modification could contribute to the formation of a more integrated and acid-resistant hybrid layer, explaining the superior functional outcomes observed in the RI + BG group. Future studies specifically designed to monitor the interfacial pH transients and characterize the etched surface morphology following BAG pretreatment are warranted to elucidate this interaction in detail.

It should be noted that while a significant statistical interaction (BG × RI) was confirmed for surface roughness and microhardness—supporting a synergistic mechanism—such interaction was not statistically significant for the LSCM morphological parameters (TF, FA, AF). In contrast, while the LSCM parameters (TF, FA, AF) also favored the RI + BG group, indicating effective pore occlusion and morphological improvement, a formal synergistic interaction was not statistically established for these static, morphological metrics. This suggests that LSCM primarily captures the lesion volume at a single timepoint, whereas the synergy detected in dynamic, functional tests (ongoing acid resistance, mechanical recovery) better reflects the enhanced biochemical stabilization critical for clinical longevity. These mechanistic insights into synergy and interaction are further supported by the detailed chemical and morphological outcomes. Thus, the term ‘synergistic’ in this study refers specifically to those functional outcomes where the combined effect exceeded the sum of individual effects, whereas the morphological improvements may be better described as ‘complementary’ or ‘additive’.

The primary limitation of this study lies in its in vitro design, which cannot fully replicate the complex oral environment, including the dynamics of saliva, oral microbiota, and masticatory forces. Nevertheless, the improved surface properties and acid resistance observed suggest that this combined approach offers a potential minimally invasive treatment for early WSLs, with enhanced clinical durability. Future studies are therefore warranted to validate its performance under real oral conditions, evaluate aesthetic outcomes and patient acceptance, and ultimately facilitate its translation into routine preventive care.

## Conclusion

Within the limitations of this in vitro study, the combination of bioactive glass and resin infiltration results in significantly superior remineralization and resistance to secondary demineralization of orthodontic white spot lesions compared to the individual use of either material. Statistical analysis provided evidence of synergistic interactions for key outcomes related to surface integrity, mechanical strength, and chemical resistance. This combined approach, supported by evidence of synergy in functional properties, shows promising potential for clinical application in managing WSLs. Future long-term in vivo studies are warranted to confirm these findings in a dynamic oral environment.

## Data Availability

The datasets used and/or analyzed during the current study are available from the corresponding author on reasonable request.
